# Burden of smoking on disease-specific mortality, DALYs, costs: the case of a high-income European country

**DOI:** 10.1186/s12889-023-15535-9

**Published:** 2023-04-14

**Authors:** Renato Farcher, Maria Eleni Syleouni, Linda Vinci, Renato Mattli

**Affiliations:** Winterthur Institute of Health Economics, Zurich University of Applied Sciences, Gertrudstrasse 15, Winterthur, 8401 Switzerland

**Keywords:** Chronic disease, Burden of disease, Cost of illness, Smoking

## Abstract

**Background:**

Smoking is a major risk factor for chronic diseases causing early death and disability. Smoking prevalence over the past 25 years has remained high in Switzerland. Evidence about the burden of disease and cost of illness attributable to smoking can support tobacco control. The aim of the present paper is to quantify from a societal perspective the mortality, disability-adjusted life years (DALYs), medical costs and productivity losses attributable to smoking in Switzerland in 2017.

**Methods:**

Smoking attributable fractions (SAFs) were calculated based on the prevalence of current and former active smoking in the latest Swiss Health Survey from 2017 and relative risks from the literature. The SAFs were then multiplied with the number of deaths, DALYs, medical costs and productivity losses in the total population.

**Results:**

In the Swiss population in 2017 smoking accounted for 14.4% of all deaths, for 29.2% of the deaths due to smoking-related diseases, 36.0% of the DALYs, 27.8% of the medical costs and 27.9% of productivity losses. Total costs amounted to CHF 5.0 billion which equals CHF 604 per capita per year. The highest disease burden in terms of mortality and DALYs attributable to smoking was observed for lung cancer and chronic obstructive pulmonary disease (COPD), whereas the highest cost of illness in terms of medical costs was observed for coronary heart diseases and lung cancer and in terms of productivity losses for COPD and coronary heart diseases. Sex and age group differences were found.

**Conclusions:**

We provide an estimate of the burden of smoking on disease-specific mortality, DALYs, medical costs and productivity losses in Switzerland that could be prevented through evidence-based tobacco prevention and control policies as well as regular monitoring of tobacco consumption.

**Supplementary Information:**

The online version contains supplementary material available at 10.1186/s12889-023-15535-9.

## Background

In 2015 smoking was the second-leading risk factor for early death and disability in Central Europe and amongst the ten largest contributors to global disability-adjusted life years (DALYs) [1]. The World Health Organization (WHO) reported that smoking prevalence of adult smokers in high income countries was higher in 2007 compared to 2017 and had been reduced from 27 to 21% [2].

In Switzerland, a high-income country in Central Europe, smoking prevalence over the past 25 years has remained high in both sexes, surpassing 30% in men and 20% in women [3]. In 2004, Switzerland signed the WHO Framework Convention on Tobacco Control (WHO FCTC) [4] but besides Andorra, Liechtenstein and Monaco, Switzerland is the only country in Europe that has not yet ratified it [5]. With a health care system currently amongst the best and most expensive in the world [6, 7], Switzerland’s provisional total health care expenditure in 2017 was estimated at 82.5 billion Swiss francs (CHF), 12.3% of the country’s gross domestic product (GDP) [8]. Being able to quantify the burden of smoking on the population by sex and age group can better inform decision makers on the current situation and prioritize the distribution of healthcare resources.

DALYs, mortality and health expenditures are considered appropriate measures for quantifying the burden of smoking on society. DALYs quantify the burden of mortality and morbidity associated with diseases, by combining the years of life lost due to premature mortality (YLL) with the years lived with disability (YLD). Hence, one DALY corresponds to one year of healthy life lost due to the disease[9]. Disease costs comprise both of the medical costs for the treatment of the diseases and of the indirect costs due to productivity losses, such as sick leave, permanent incapacity for work or premature death.

In 2015, smoking was responsible for 6.4 million deaths worldwide [10], and the first leading risk factor in terms of DALYs for Switzerland; France; Denmark; Netherlands; United Kingdom (UK) and second leading risk factor for Germany; Italy and Austria [1]. The global economic cost of smoking attributable diseases in high income countries was found to account for 6.5% of the total health expenditure and 2.2% of the GDP [11]. To our knowledge, the health care costs and productivity losses caused by tobacco consumption were last calculated for Switzerland in 1995 [12] and the smoking-related DALYs by age group and sex have never been calculated in detail for Switzerland before.

The objective of this study was to estimate the burden of disease attributable to smoking in the Swiss population for 2017. By using well established methods, this study provides an update and an overview of the current situation on a national level. Furthermore, it facilitates the assessment of the smoking-related measures in place and encourages the design of future strategies and prioritization of further measures. This study contributes to the international literature by providing the complete burden of disease profile due to smoking of a high-income country, allowing for international comparison.

## Methods

### Design

For the estimation of the burden of smoking in Switzerland in 2017 we considered a societal perspective following a top-down approach using prevalence-based smoking attributable fractions (SAFs). SAFs can be thought as the proportional decrease in the incidence of a disease if the entire population were no longer smokers [13] or had they not been smokers in the past. This prevalence-based method has been implemented in the majority of studies [14], and is based on Levin’s formula, as described by Hanley [15].

**Fig. 1:**
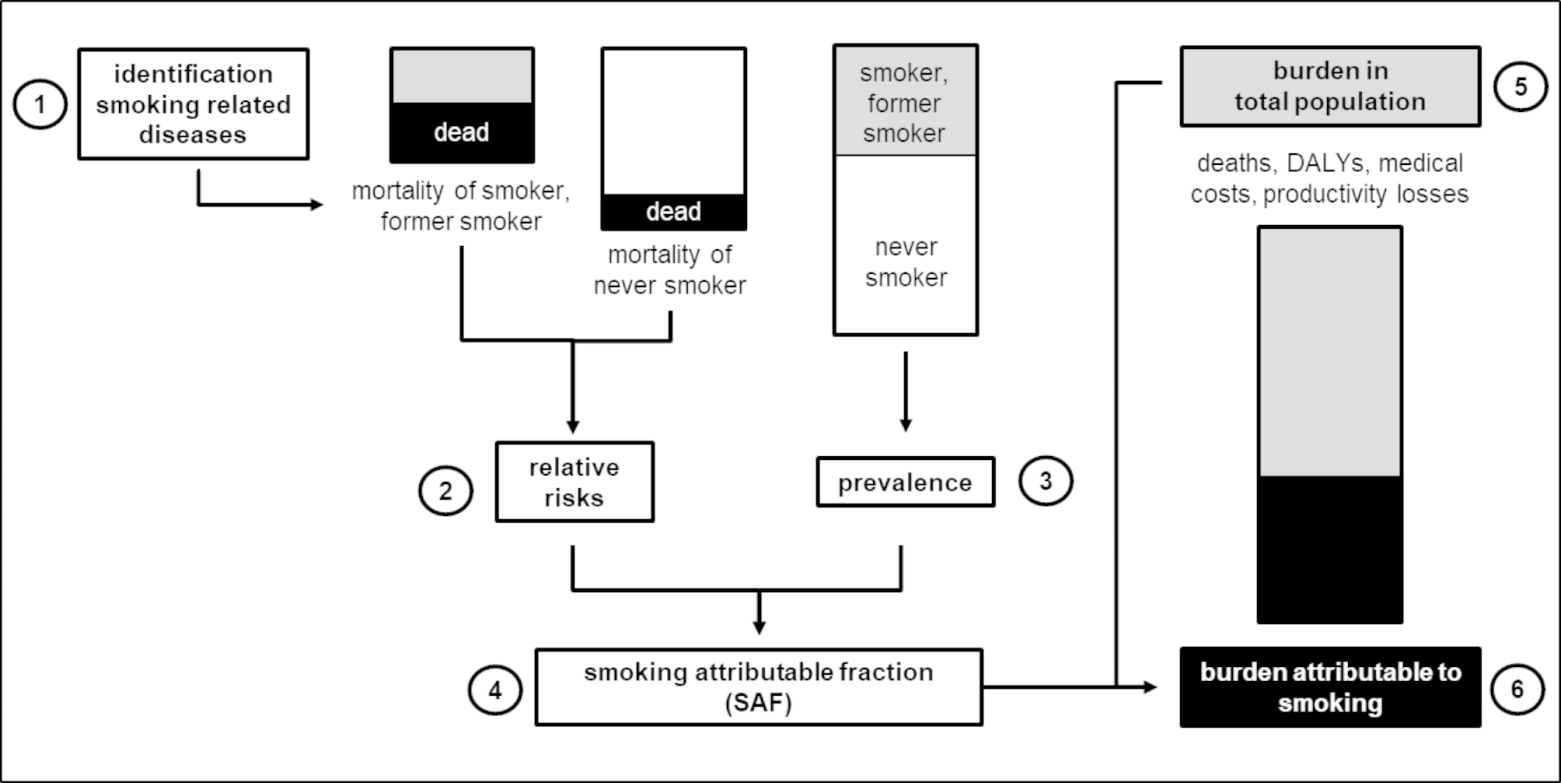
Overview of study design The burden of disease in the total population was extracted from specific data sources. Smoking related diseases were based on literature (1). The relative risks of these diseases were extracted from the General Surgeon Report 2014 (2) and combined with the prevalence of current, former and never smokers acquired from the latest Swiss Health Survey (3) for the calculation of the smoking attributable fractions (SAFs) (4). In the last step, SAFs were multiplied with mortality, disability-adjusted life years (DALYs), medical costs and productivity losses in the total population (5) to get to the burden attributable to smoking (6).

A schematic approach of the design is presented in Fig. 1. In a first step the smoking attributable diseases and their respective relative risks (RR) were identified. Then, the prevalence of smokers and former smokers for Switzerland was acquired. As next step the RR were combined with the prevalence of current and former smokers to estimate the SAFs. The SAFs were then multiplied with the number of deaths, DALYs, medical costs and productivity losses in the total population resulting in deaths, DALYs, and costs attributable to smoking. Due to data availability, we restricted our analysis to the adult population aged 35 years and older and given there is a time-lag for smoking to cause harm, the age ranges between 30 and 35 years are considered appropriate to start measuring the effects of smoking [14].

### Diseases included in the analysis and data used

For the present study the following smoking-related diseases and disease groups were selected: lung cancer; other cancers; coronary heart disease; other heart diseases; cerebrovascular disease; other vascular diseases; diabetes mellitus; pneumonia, influenza and tuberculosis; chronic obstructive pulmonary disease (COPD). Other cancers include cancers of the lip, pharynx and oral cavity, esophagus, stomach, pancreas, larynx, cervix uteri (women), kidney and renal pelvis, bladder, liver, colon and rectum, acute myeloid leukemia. Other heart diseases are comprised of rheumatic heart disease, pulmonary heart disease, and other forms of heart disease. Whereas other vascular diseases consist of atherosclerosis, aortic aneurysm, and other arterial diseases. The additional file 1 provides a detailed overview of the composition of all the included diseases.

RR of death for current versus never smokers ($R{R}_{CS\_as}$) and RR of death for former versus never smokers ($R{R}_{FS\_as}$) by smoking associated disease and age (a) and sex (s) specific, were obtained from the General Surgeon Report (GSR) 2014 [16] (see additional file 2).

The respective prevalence for each sex and age group was available from the Swiss Health Survey (SHS) [3] conducted by the Swiss Federal Statistical Office (FSO). The SHS takes place every five years and reports data for the age groups 15–24, 25–34, 35–44, 45–54, 55–64, 65–74 and older than 75 years. Currently available are self-reported smoking status data from 1992 to 2017 and for the present study, the most recent data from 2017 were used. As current smoker was defined any participant who at the time of the survey reported smoking any tobacco or nicotine product (cigarettes, cigars, cigarillos, pipe, waterpipe, electronic cigarettes, heated tobacco products) and as former smoker was defined any participant reporting to have smoked any tobacco or nicotine product regularly for more than six months in the past. The rest of the participants were defined as never smokers.

For mortality, data from the Causes of Death Statistics [17] administered by the FSO for 2017 were used. These mortality data are collected yearly and information about the age, sex and cause of death indicated by an ICD-10 code is provided for each death.

The DALYs for 2017 by disease, sex and age group were extracted for Switzerland from the Global Burden of Disease (GBD) study 2017 [18]. The GBD 2017 study is a comprehensive global collaborative research program that determines the burden of disease, injuries and risk factors. For most diseases DALYs could be assigned directly, whereas for the ones not directly available, the following groups were considered: other forms of heart disease (rheumatic heart disease, cardiomyopathy and myocarditis, arterial fibrillation and flutter, endocarditis, other cardiovascular and circulatory disease); cerebrovascular disease (stroke); arterial disease (peripheral artery disease); pneumonia, influenza, tuberculosis (lower respiratory infections) For atherosclerosis no DALYs could be identified.

Medical costs and indirect costs as a result of productivity losses were derived mainly from the cost-of-illness study of non-communicable diseases in Switzerland [19]. In this study though, there were no costs available for acute myeloid leukemia; atherosclerosis; other arterial diseases; and pneumonia. Therefore, costs of these diseases were estimated based on the study by Dieleman et al. [20], where the medical costs in the United States (US) of 155 diseases for the year 2013 were estimated systematically as part of the GBD Study. For the medical costs and productivity losses there were no data available for influenza, tuberculosis, aortic aneurysm and dissection. The costs of other forms of heart disease have not been considered separately because we assume these costs are included in the cost of coronary heart disease. For pneumonia, influenza, tuberculosis, the group “lower respiratory diseases” were used for medical costs [20]. For the extrapolation of the costs to the year 2017, the official statistics on the costs and financing of the health system of the FSO were used [21].

### Analysis

The analysis was performed in two steps. First, we estimated the SAFs for each disease, sex and age group using the polytomous exposure case approach based on the prevalence as described by Hanley [15]. Prevalence data of current smokers ${P}_{CS}$ and former smokers ${P}_{FS}$ were used, although the prevalence for the age group 35–54 was not directly available and was thus estimated by the combined estimates for the age groups 35–44 and 45–54.

The following formula was applied:

$$\begin{array}{c}SA{F_{as}}\left( \% \right) = 100\\* \frac{{\left[ {{P_{FS - as}} * \left( {R{R_{FS - as}} - 1} \right) + {P_{CS - as}} * \left( {R{R_{CS - as}} - 1} \right)} \right]}}{{\left[ {{P_{FS - as}} * \left( {R{R_{FS - as}} - 1} \right) + {P_{CS - as}} * \left( {R{R_{CS - as}} - 1} \right) + 1} \right]}}\end{array}$$

where the “as” index denotes age group and sex.

Second, given that we followed a disease specific approach, we calculated for smoking the attributable total mortality, the total DALYs, as well as the total medical and indirect costs for 2017 in Switzerland.

For the cost estimation, we used the disease specific share of smoking-attributed deaths on the total number of deaths in the year 2017 as the SAF, since detailed cost data on age group and sex is not available. The total cost of the diseases not included in the cost-of-illness study of non-communicable diseases in Switzerland [19] were first divided by the US prevalence according to GBD [18] to receive the per-capita costs in US dollars for 2013. Wieser et al. [22] showed that the cost per capita between Switzerland and the US does not differ significantly. The costs were then converted into Swiss francs according to the exchange rate published by the Swiss National Bank [23]. Subsequently, per capita costs per person were extrapolated to 2017 according to the increase in per capita health care costs. To obtain the total cost of acute myeloid leukemia, the per capita cost was multiplied by the prevalence according to the National Institute of Cancer Epidemiology and Registration. For the total costs of the other three diseases, per capita costs were multiplied by the prevalence for Switzerland for 2017 according to GBD [18].

For the analysis the software STATA 2015 and Microsoft Excel 2016 were used.

## Results

We estimated the SAFs, mortality, DALYs, and costs attributable to smoking for lung cancer; other cancers; coronary heart disease; other heart diseases; cerebrovascular diseases; other vascular diseases; diabetes mellitus; pneumonia, influenza and tuberculosis; and COPD. Our results suggest that for both men and women the highest SAFs irrespective of the age group are observed in lung cancer and COPD. For the age group of 65–74 years, the SAF values for lung cancer reach 89.5% for men and 83.5% for women, whilst for COPD they reach 89.9% for men and 90.8% for women (Tables 1 and 2).

**Table 1:** Smoking attributable fraction, mortality and DALYs for men in Switzerland in 2017

Disease Groups	Age groups (years)													
	35–54			55–64			65–74			≥ 75			Total	
	**SAF**	**Mortality**	**DALYs**	**SAF**	**Mortality**	**DALYs**	**SAF**	**Mortality**	**DALYs**	**SAF**	**Mortality**	**DALYs**	**Mortality** (%)	**DALYs** (%)
**Total malignant neoplasms**													**2845 (48.0%)**	**55,945 (49.0%)**
Lung cancer	0.838	113	4535	0.865	308	9992	0.895	609	12,654	0.840	706	8010	1736 (86.3%)	35,191 (86.6%)
Other cancers^a^	0.245	62	2568	0.261	161	4920	0.329	369	7710	0.268	517	5556	1109 (28.3%)	20,754 (28.2%)
**Total cardiovascular diseases and diabetes mellitus**													**2048 (22.5%)**	**48,546(28.0%)**
Coronary heart diseases	0.533	91	4831	0.429	141	5613	0.379	249	6876	0.215	591	7774	1072 (27.5%)	25,094 (32.8%)
Other heart diseases^b*^	0.326	30	983	0.378	63	1522	0.279	105	1856	0.133	263	1438	461 (17.6%)	5799 (23.6%)
Cerebrovascular diseases*	0.326	14	842	0.378	26	1337	0.252	53	1575	0.104	119	1563	212 (14.5%)	5317 (19.4%)
Other vascular diseases^c*^	0.326	6	182	0.378	12	423	0.641	59	1280	0.453	160	1324	237 (47.8%)	3209 (48.6%)
Diabetes mellitus*	0.326	8	3028	0.378	22	3393	0.253	23	2399	0.028	13	307	66 (10.2%)	9127 (23.6%)
**Total respiratory diseases**													**1100 (60.0%)**	**29,701 (72.5%)**
Pneumonia, Influenza, Tuberculosis*	0.587	12	393	0.836	17	814	0.373	25	622	0.218	143	1135	197 (25.8%)	2964 (34.8%)
COPD*	0.587	18	2304	0.836	65	4624	0.899	201	8028	0.843	619	11,138	903(84.7%)	26,094(82.6%)
**Total (% share of total for the specific age group)**		**354** **(44.9%)**	**19,666** **(43.7%)**		**815** **(47.2%)**	**32,638** **(48.2%)**		**1693** **(48.1%)**	**43,000** **(47.4%)**		**3131** **(28.9%)**	**38,245 (30.9%)**	**5993** **(35.5%)**	**133,549 (40.8%)**

SAF: Smoking attributable fraction, DALYs: Disability adjusted life years, COPD: chronic obstructive pulmonary disease, ^a^Other cancers include cancers of the lip, pharynx and oral cavity, esophagus, stomach, pancreas, larynx, cervix uteri (women), kidney and renal pelvis, bladder, liver, colon and rectum, acute myeloid leukemia, ^b^Other heart diseases include rheumatic heart disease, pulmonary heart disease, and other forms of heart disease, ^c^Other vascular diseases include atherosclerosis, aortic aneurysm, and other arterial diseases * For these diseases the same SAF were chosen for the age groups 35–54, 55–64, 65–74 due to the RR availability. All numbers are rounded.

**Table 2:** Smoking attributable fraction, mortality and DALYs for women in Switzerland in 2017

Disease Groups	Age groups (years)													
	35–54			55–64			65–74			≥ 75			Total	
	**SAF**	**Mortality**	**DALYs**	**SAF**	**Mortality**	**DALYs**	**SAF**	**Mortality**	**DALYs**	**SAF**	**Mortality**	**DALYs**	**Mortality** (%)	**DALYs** (%)
**Total malignant neoplasms**													**1400 (35.3%)**	**28,680 (38.7%)**
Lung cancer	0.776	66	3260	0.842	208	6659	0.835	357	7098	0.724	390	4008	1021 (78.7%)	21,025 (80.4%)
Other cancers^a^	0.105	17	766	0.249	74	2486	0.189	118	2576	0.107	170	1827	379 (14.2%)	7655 (16.0%)
**Total cardiovascular diseases and diabetes mellitus**													**1278 (13.0%)**	**22,086 (16.2%)**
Coronary heart diseases	0.556	16	1030	0.371	23	1177	0.335	72	2326	0.148	429	5418	540 (16.9%)	9951 (20.5%)
Other heart diseases^b*^	0.269	9	600	0.206	8	468	0.174	32	722	0.106	349	1392	398 (11.3%)	3182 (14.6%)
Cerebrovascular diseases*	0.269	6	655	0.206	10	601	0.206	28	1064	0.064	118	1340	162 (7.9%)	3660 (11.6%)
Other vascular diseases^c*^	0.269	2	49	0.206	2	73	0.552	22	456	0.349	132	807	158 (36.3%)	1385 (37.7%)
Diabetes mellitus*	0.269	1	1594	0.206	4	1124	0.141	5	952	0.019	10	238	20 (3.6%)	3908 (12.8%)
**Total respiratory diseases**													**830 (45.0%)**	**21,102 (63.6%)**
Pneumonia, Influenza, Tuberculosis*	0.608	5	207	0.745	13	339	0.161	6	151	0.104	87	557	111 (12.3%)	1254 (17.7%)
COPD *	0.608	5	1857	0.745	46	2989	0.908	179	6051	0.725	489	8951	719 (76.3%)	19,848 (76.1%)
**Total (% share of total for the specific age group)**		**127 (35.1%)**	**10,018** **(36.4%)**		**388 (48.4%)**	**15,916** **(43.6%)**		**819** **(43.1%)**	**21,396** **(40.0%)**		**2174 (17.3%)**	**24,538 (19.5%)**	**3508** **(22.4%)**	**71,868** **(29.5%)**

SAF: Smoking attributable fraction, DALYs: Disability adjusted life years, COPD: chronic obstructive pulmonary disease, ^a^Other cancers include cancers of the lip, pharynx and oral cavity, esophagus, stomach, pancreas, larynx, cervix uteri (women), kidney and renal pelvis, bladder, liver, colon and rectum, acute myeloid leukemia, ^b^Other heart diseases include rheumatic heart disease, pulmonary heart disease, and other forms of heart disease, ^c^Other vascular diseases include atherosclerosis, aortic aneurysm, and other arterial diseases * For these diseases the same relative risks for the age groups 35–54, 55–64, 65–74 were used. ** See indication above (*). All numbers are rounded.

In 2017, smoking accounted for 14.4% of all deaths in the Swiss population older than 35 years (18.9% in men and 10.3% in women). Smoking contributed 5993 deaths of men and 3508 deaths of women older than 35 years, corresponding to 29.2% of all deaths due to smoking-related diseases in the Swiss population in this age group (35.5% in men and 22.4% in women) (Tables 1, 2 and 3). Especially in lung cancer and COPD, about 80% of the total deaths for both sexes were attributable to smoking (Table 3). In men older than 35 years, a high number of deaths attributable to smoking is observed for lung cancer (86.3%); COPD (84.7%); other cancers (28.3%) and coronary heart diseases (27.5%) (Table 1). In women of the same age group a high number of deaths attributable to smoking is observed for lung cancer (78.7%); COPD (76.3%) and coronary heart diseases (16.9%) (Table 2). We estimated that in men, smoking was responsible for 47.2% and 48.1% of all deaths due to smoking-related diseases in the age groups 55–64 and 65–74 respectively, and for 28.9% of all deaths in the age group above 75 years old. Likewise, in women smoking was responsible for 48.4% of all deaths due to smoking-related diseases in the age group 55–64 and 43.1% of all deaths in the age group 65–74, while only 17.3% of deaths in the oldest age group were due to smoking. Nonetheless, in the older age groups larger numbers of deaths occur in general, thus a larger number of smoking attributable deaths is observed.

**Table 3:** Burden of smoking for men and women 35 + years of age in Switzerland in 2017

Disease Groups						
	Smoking Attributable Mortality (%)	Smoking Attributable DALYs (%)	Smoking Attributable YLL (%)	Smoking Attributable YLD (%)	Smoking Attributable Medical Costs in Mio CHF (%)	Smoking Attributable Indirect Costs in Mio CHF (%)
**Total malignant neoplasms**	**42.9%**	**44.9%**	**45.4%**	**32.5%**	**38.9%**	**32.1%**
Lung cancer	2757 (83.3%)	56,218 (84.2%)	55,362 (84. 2%)	855 (83.9%)	695.8 (23.0%)	227.2 (11.2%)
Other cancers^a^	1488 (22.6%)	28,408 (23.4%)	27,169 (23.4%)	1239 (22.9%)	511.5 (16.9%)	330.7 (16.3%)
**Total cardiovascular diseases and diabetes mellitus**	**17.6%**	**22.8%**	**23.7%**	**20.9%**	**16.3%**	**15.1%**
Coronary heart diseases	1612 (22.7%)	35,045 (28.0%)	32,415 (27.9%)	2630 (30.6%)	726.9 (24.1%)	480.3 (23.6%)
Other heart diseases^b*^	859 (14.0%)	8981 (19.4%)	5227 (18.3%)	3754 (21.2%)		
Cerebrovascular diseases*	373 (10.6%)	8977 (15.2%)	5843 (14.2%)	3155 (17.5%)	257.1 (8.5%)	119.1 (5.9%)
Other vascular diseases^c*^	395 (42.4%)	4595 (44.7%)	4154 (44. 6%)	441 (46.4%)	42.9 (1.4%)	
Diabetes mellitus*	**88 (7.0%)**	**13,036 (18.8%)**	**1809 (13.7%)**	**11,226 (20.0%)**	**70.3 (2.3%)**	**95.5 (4.7%)**
**Total respiratory diseases**	**52.5%**	**68.5%**	**61.9%**	**77.9%**	**68.2%**	**80.8%**
Pneumonia, Influenza, Tuberculosis*	308 (18.5%)	4219 (27.0%)	4192 (26.9%)	27 (38.0%)	39.0 (1.3%)	
COPD*	1623 (80.8%)	46,193 (79. 7%)	22,553 (81.5%)	23,640 (78.0%)	678.3 (22.4%)	781.8 (38.4%)
**Total (% share of total of smoking-related diseases)**	**9503 (29.2%)**	**205,672 (36.0%)**	**158,724 (36.6%)**	**46,947 (34.0%)**	**3021.8 (27.8%)**	**2034.6 (27.9%)**

DALYs (%): Disability adjusted life years (% share of total DALYs for the specific disease), YLL (%): Years of life lost (% share of total YLL for the specific diseases, YLD (%): Years lived with disability (% share of total YLD for the specific diseases), ^a^Other cancers includes cancers of the lip, pharynx and oral cavity, esophagus, stomach, pancreas, larynx, cervix uteri (women), kidney and renal pelvis, bladder, liver, colon and rectum, acute myeloid leukemia, ^b^Other heart disease includes rheumatic heart disease, pulmonary heart disease, and other forms of heart disease, ^c^Other vascular diseases includes atherosclerosis, aortic aneurysm, and other arterial diseases, * For these diseases the same relative risks for the age groups 35–54, 55–64, 65–74 were used. All numbers are rounded.

Regarding DALYs, in Switzerland smoking was responsible for 36.0% of all DALYS due to smoking-related diseases in 2017. For the age groups below 75 years old, the DALYs attributable to smoking were more than 44% in men and more than 35% in women. The majority of DALYs attributable to smoking in men arise from lung cancer, COPD, coronary heart diseases and other cancers, whilst in women primarily arise from lung cancer and COPD (Tables 1 and 2). Decomposing DALYs in YLL and YLD, 77.2% of DALYs are from YLL and 22.8% from YLD.

Based on the estimated SAFs for the above-mentioned diseases, the total costs for 2017 attributable to smoking in Switzerland result to more than CHF 5056.4 million, CHF 604 per capita per year. The medical costs account for 3.8% of the total health expenditures. The medical costs sum up to CHF 3021.8 million, and the smoking attributable diseases inferring the highest medical costs with decreasing order are coronary heart diseases (24.1%); lung cancer (23.0%); COPD (22.4%) and other cancers (16.9%). The indirect costs due to productivity losses sum up to CHF 2034.6 million and the diseases with the highest contributions to the indirect costs are COPD (38.4%); coronary heart diseases (23.6%); other cancers (16.3%).

## Discussion

In our study we estimated the burden of smoking in 2017 for the Swiss population older than 35 years for lung cancer; other cancers; coronary heart diseases; other heart diseases; cerebrovascular diseases; other vascular diseases; diabetes mellitus; pneumonia, influenza, tuberculosis; and COPD. For those diseases smoking accounted for 29.2% of the deaths, 36.0% of the DALYs, 27.8% of the medical costs, and 27.9% of the indirect costs from productivity losses. In total, smoking accounted for 14.4% of all deaths in the Swiss population older than 35 years in 2017 (18.9% in men and 10.3% in women).

Our results are in line with previous studies although comparisons should be made with caution due to methodological differences, data sources and demographics. Comparing Switzerland with its neighboring countries, the prevalence of daily smokers in Switzerland for 2015 (21.1%) was comparable; Austria (23.5%), Germany (24.4%), France (27.4%) and Italy (19.8%) [24].

Regarding smoking attributable mortality, in our study smoking accounted for 29.2% of the deaths due to smoking-related diseases, 35.5% among men and 22.4% among women. These proportions are higher than those estimated in UK for the year 2005 [25] (18.6% of deaths, 27.2% of male deaths, 10.5% of female deaths) and those in Germany for the year 2013 [26] (14% of deaths). Our study results of smoking attributable cancer deaths, 48.0% of all deaths among men and 35.3% deaths among women, are in line with the estimates from the USA for the year 2011 (44–48%, 32–37%, respectively) [27]. The contribution of all smoking attributable deaths from cardiovascular diseases and diabetes in the present study, 22.5% among men and 13.0% among women, was lower compared to the estimates from the USA in 2011 (27–32%, 31–38%, respectively) [27].

Our estimations on the proportion of deaths attributable to smoking for major non-communicable diseases on both sexes are in agreement with those published for Europe in the 2004 WHO report on global mortality [28]. More specifically our estimation for both sexes attributes 83.3% of lung cancer deaths and 80.8% of COPD deaths to smoking. In the 2004 WHO report the estimates are 82% and 70% respectively [28]. For other respiratory diseases the proportion of deaths attributable to smoking for Switzerland was estimated at 18.5%, while in the 2004 WHO report it is 18% [28]. In cerebrovascular diseases our estimate is 10.6% and the corresponding estimate in the 2004 WHO report is 6% [28]. It should be noted though, that except for the different year, the formula used by the WHO for the estimation of SAFs was different than the one we used for our estimations [28].

Regarding DALYs, a study from UK estimated that in 2002 smoking was responsible for 85% of lung cancer DALYs, 68% of the COPD DALYs and 22% of the cardiovascular diseases DALYs [25]. In our study the respective numbers are 84.2%, 79.7% and 22.8%. The age of the population though is different between the two studies as well as the methodology used for the calculation of SAFs.

Concerning costs, Goodchild and colleagues reported findings from various studies of smoking attributable costs for various countries [11]. The SAF in percentage of health care expenditures for Germany, France and Italy was reported between 3.2% and 6.3% [11]. However, we estimated a SAF in percentage of health care expenditures of 3.8% for Switzerland whereas Goodchild et al. [11] reported 3.4%.

One strength of our study is the use of a solid methodology to quantify the burden of smoking, which subsequently allows the comparison of the achievements of tobacco control measures in Switzerland with those of other countries. The novelty in our study is the use of the polytomous exposure approach, where both current and former-smoker relative risks are considered. Our results translate to the fraction of all cases of current smokers and former smokers that would not have occurred if exposure had not occurred, applying on non-passive smokers. Most of the publications consider only the current smokers for the estimation of the SAF, whereas the former-smokers and their contribution to the burden of disease attributable to smoking is neglected. Moreover, our analysis includes a younger age group, not taken into account by many studies and for which our results yielded that the proportion of deaths due to smoking reaches 29%. Finally, our study provides a complete picture on the burden of smoking on society by age group and sex whenever possible, allowing the policy makers to assess probable benefits and targeted interventions.

One of the limitations of our study is the use of RRs from a different population. We used RRs from the GSR study, which is based on US population (white race 94%) [29], under the assumption that the Swiss and US populations are similar in characteristics, disease latency, as well as the disease mechanisms. Another limitation is the absence of confidence intervals for our estimates due to the lack of data; the confidence intervals would have allowed us to quantify the uncertainty of our results and comparison with other studies. Finally, the latency from smoking initiation to the onset of the disease was not taken into account, and given the smoking-related diseases considered in this study, the burden of smoking might be underestimated.

## Conclusions

We provide an estimate of the burden of smoking on disease-specific mortality, DALYs, medical costs and productivity losses in Switzerland that could be prevented through evidence-based tobacco prevention and control policies as well as regular monitoring of tobacco consumption. Our quantitative findings on the burden of smoking could inform public health interventions to reduce the prevalence of smoking.

## Electronic supplementary material

Below is the link to the electronic supplementary material.

Supplementary Material 1

## Data Availability

The datasets used and/or analysed during the current study are available in the Swiss federal statistical office repository, https://www.bfs.admin.ch/bfs/en/home/statistics/health.html and in the Global Burden of Disease study repository of the Institute for Health Metrics and Evaluation of the University of Washington, https://vizhub.healthdata.org/gbd-results/.

## Abbreviations

DALYs: Disability-adjusted life years
WHO: World Health Organization
WHO FCTC: World Health Organization Framework Convention on Tobacco Control
CHF: Swiss francs
GDP: Gross domestic product
YLL: Years of life lost
YLD: Years lived with disability
UK: United Kingdom
SAFs: Smoking attributable fractions
RR: Relative risks
COPD: Chronic obstructive pulmonary disease
GSR: General Surgeon Report
SHS: Swiss Health Survey
FSO: Swiss Federal Statistical Office
GBD: Global Burden of Disease
US: United States
