# Supplementary material for: Burden of smoking on disease-specific mortality, DALYs, costs: the case of a high-income European country
Source: BMC Public Health. 2023 Apr 14;23:698. doi: 10.1186/s12889-023-15535-9 (PMC10103371; doi:10.1186/s12889-023-15535-9)
Supplement: Supplementary file 1 — Supplementary Material 1 [file 12889_2023_15535_MOESM1_ESM.docx]

**Supplementary material**

*Additional file 1: Included smoking-related diseases*

| Malignant neoplasm | | |  |
| --- | --- | --- | --- |
|  | Lung cancer | |  |
|  | Other cancers | |  |
|  |  | Lip, pharynx and oral cavity | |
|  |  | Esophagus | |
|  |  | Stomach | |
|  |  | Colon and rectum | |
|  |  | Liver | |
|  |  | Pancreas | |
|  |  | Larynx | |
|  |  | Bladder | |
|  |  | Kidney and renal pelvis | |
|  |  | Cervix uteri | |
|  |  | Acute myeloid leukemia | |
| Cardiovascular diseases | | |  |
|  | Coronary heart diseases | |  |
|  | Other heart diseases | |  |
|  |  | Rheumatic heart disease | |
|  |  | Pulmonary heart disease | |
|  |  | Other forms of heart disease | |
|  | Cerebrovascular diseases | |  |
|  | Other vascular diseases | |  |
|  |  | Atherosclerosis | |
|  |  | Aortic aneurysm | |
|  |  | Other arterial diseases | |
| Diabetes mellitus | | |  |
| Respiratory diseases | | |  |
|  | Pneumonia, Influenza, Tuberculosis | |  |
|  | COPD | |  |

*Additional file 2: Relative risks for disease specific death by sex, smoking status and age group*

|  | **Men** | | | | | | | | **Women** | | | | | | | |
| --- | --- | --- | --- | --- | --- | --- | --- | --- | --- | --- | --- | --- | --- | --- | --- | --- |
|  | **Current smokers** | | | | **Former smokers** | | | | **Current smokers** | | | | **Former smokers** | | | |
|  | **35-54** | **55-64** | **65-74** | **>=75** | **35-54** | **55-64** | **65-74** | **>=75** | **35-54** | **55-64** | **65-74** | **>=75** | **35-54** | **55-64** | **65-74** | **>=75** |
| Lung cancer | 14.33 | 19.03 | 28.29 | 22.51 | 4.40 | 4.57 | 7.79 | 6.46 | 13.30 | 18.95 | 23.65 | 23.08 | 2.64 | 5.00 | 6.80 | 6.38 |
| Other cancers | 1.74 | 1.86 | 2.35 | 2.18 | 1.36 | 1.31 | 1.49 | 1.46 | 1.28 | 2.08 | 2.06 | 1.93 | 1.24 | 1.28 | 1.26 | 1.27 |
| Coronary heart diseases | 3.88 | 2.99 | 2.76 | 1.98 | 1.83 | 1.52 | 1.58 | 1.32 | 4.98 | 3.25 | 3.29 | 2.25 | 2.23 | 1.21 | 1.56 | 1.42 |
| Other heart diseases^a^ | 2.40 | 2.51 | 2.22 | 1.66 | 1.07 | 1.51 | 1.32 | 1.15 | 2.44 | 1.98 | 1.85 | 1.75 | 1.00 | 1.10 | 1.29 | 1.32 |
| Cerebrovascular disease^a^ | 2.40 | 2.51 | 2.17 | 1.48 | 1.07 | 1.51 | 1.23 | 1.12 | 2.44 | 1.98 | 2.27 | 1.70 | 1.00 | 1.10 | 1.24 | 1.10 |
| Other vascular diseases^a*^ | 2.40 | 2.51 | 7.25 | 4.93 | 1.07 | 1.51 | 2.20 | 1.72 | 2.44 | 1.98 | 6.81 | 5.77 | 1.00 | 1.10 | 2.26 | 2.02 |
| Diabetes mellitus^a^ | 2.40 | 2.51 | 1.50 | 1.00 | 1.07 | 1.51 | 1.53 | 1.06 | 2.44 | 1.98 | 1.54 | 1.10 | 1.00 | 1.10 | 1.29 | 1.06 |
| Pneumonia, influenza, tuberculosis^b^ | 4.47 | 15.2 | 2.58 | 1.62 | 2.22 | 3.98 | 1.62 | 1.42 | 6.43 | 9.00 | 1.75 | 2.06 | 1.85 | 4.84 | 1.28 | 1.21 |
| Chronic obstructive pulmonary disease^b^ | 4.47 | 15.2 | 29.69 | 23.01 | 2.22 | 3.98 | 8.13 | 6.55 | 6.43 | 9.00 | 38.89 | 20.96 | 1.85 | 4.84 | 15.72 | 7.06 |
| Source: General Surgeon Report 2014  Other cancers include of cancers of the lip, pharynx and oral cavity, esophagus, stomach, pancreas, larynx, cervix uteri (women), kidney and renal pelvis, bladder, liver, colon and rectum and acute myeloid leukemia  Other heart diseases include rheumatic heart disease, pulmonary heart disease and other form of heart disease  Other vascular diseases include atherosclerosis, aortic aneurysm, and other arterial diseases  ^a^ For the age groups 35-54 and 55-64 years a relative risk for cardiovascualar diseases compromised of other heart disease, cerebrovascular disease, other vascular diseases, and diabetes mellitus was used  ^b^ For the age groups 35-54 and 55-64 years a relative risk compromised of pneumonia, influenza, tuberculosis and chronic obstructive pulmonary disease was used | | | | | | | | | | | | | | | | |
